# Hydrogels as delivery systems for spinal cord injury regeneration

**DOI:** 10.1016/j.mtbio.2021.100093

**Published:** 2021-01-22

**Authors:** D. Silva, R.A. Sousa, A.J. Salgado

**Affiliations:** a Life and Health Sciences Research Institute (ICVS), School of Medicine, University of Minho, Campus de Gualtar, 4710-057, Braga, Portugal; b ICVS/3B's—PT Government Associate Laboratory, 4710-057/4805-017, Braga/Guimarães, Portugal; c Stemmatters, Biotecnologia e Medicina Regenerativa SA, 4805-017, Guimarães, Portugal

**Keywords:** Spinal cord injury, Hydrogels, Delivery system, Regeneration, Therapeutic agent

## Abstract

Spinal cord injury is extremely debilitating, both at physiological and psychological levels, changing completely the patient's lifestyle. The introduction of biomaterials has opened a new window to develop a therapeutic approach to induce regeneration after injury due to similarities with extracellular matrix. Particularly, hydrogels have the ability to support axonal growth and endogenous regeneration. Moreover, they can also act as potential matrixes in which to load and deliver therapeutic agents at injury site. In this review, we highlight some important characteristics to be considered when designing hydrogels as delivery systems (DS), such as rheology, mesh size, swelling, degradation, gelation temperature and surface charge. Additionally, affinity-based release systems, incorporation of nanoparticles, or ion-mediated interactions are also pondered. Overall, hydrogel DS aim to promote a sustained, controlled and prolonged release at injury site, allowing a targeted oriented action of the therapeutic agent that will be used.

## Graphical abstract

**Figure:**
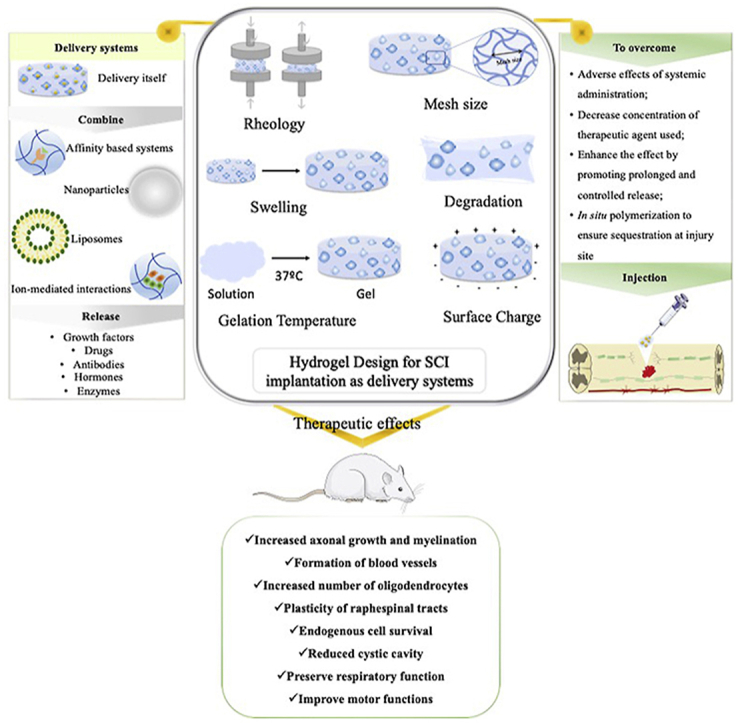

## 1 Overview of spinal cord injury

Spinal cord injury (SCI) was firstly described by Edwin Smith in an ancient Egyptian papyrus [1]. At that time, it was possible to study injuries in the central nervous system (CNS) due to the high number of accidents caused by construction of Egyptian pyramids. In this document they describe SCI as a loss of function and sensitivity below the level of injury. Moreover, they described it as “an ailment not to be treated” [2]. Later on, Hippocrates associated other complications to this condition, such as constipation, dysuria, skin problems, and edema, which could lead to patient's death [2].

The first big improvement in SCI patient care came as consequence of the drastically high numbers of deaths caused by SCI during World War I, due to low level of palliative cares provided to patients. As a consequence, a significant effort was made to establish multidisciplinary care centers and recruiting specialist to treat and follow civilians and military in the World War II [3,4]. As a result, an improvement in life expectancy of patients with SCI in the last decades was achieved, disclosing levels slightly lower than the average rate for non-SCI individuals. However, there are some facts than can influence life expectancy, namely severity of injury, age, gender, and also the fact that low-income countries have higher mortality rates due to the lack of economic resources available for medical care [5,6].

### 1.1 Epidemiology

In a study performed by the Global Burden of Diseases (GBDs) in 2016, the incidence of new SCI cases was 0.93 millions with a prevalence of 27 millions, being higher in high-income countries (0.29 millions) as USA or Canada, compared with low income (0.08 millions) such as Zimbabwe, and Mozambique [7]. Curiously, the incidence of SCI is also different among genders in these regions. In high-income countries, men are slightly more affected than women, specially between 20 and 40 years old, whereas in low-income countries, there is a high incidence of men affected, mainly because most of women stay at home to take care of family [6,7]. This is also related with the direct causes of SCI, which mostly are falls, traffic accidents and sport activities. In some regions of North Africa and Middle East terrorism and violence are the main cause of traumatic injuries [7,8]. Regarding non-traumatic injuries, the principal causes are associated with cord infarction, transverse myelitis, spinal abscess, or spinal canal stenosis [9]. SCI can also affect different functions of the body, depending on the region it occurs. Among all, around 50% of injuries occur at cervical level with an impact in respiratory functions, movement of arms, and all functions below the neck. Overall, these are the most severe and also the worst regarding survival and life expectancy. Injuries at thoracic, lumbar, or sacral regions are less frequent with a better prognosis of survival. Normally, these injuries compromise the control of abdominal muscles, loss of bladder and bowel control, as well as sexual function, hips, and legs movement [10]. Both can be complete, with a total loss of function below the injury, or incomplete, where only one part of the spinal cord (SC) is affected, in which some function bellow the injury level can happen [11].

The visible image of a patient with a SCI is a wheelchair-dependent person; however, there are far more than motor and physiological consequences. For instance, patients with SCI are prone to have depression, anxiety, sleep disturbances, and autonomic dysreflexia [12], which frequently lead to an increase in suicide among SCI people [13].

### 1.2 Pathophysiology and barriers for regeneration

**Fig. 1:**
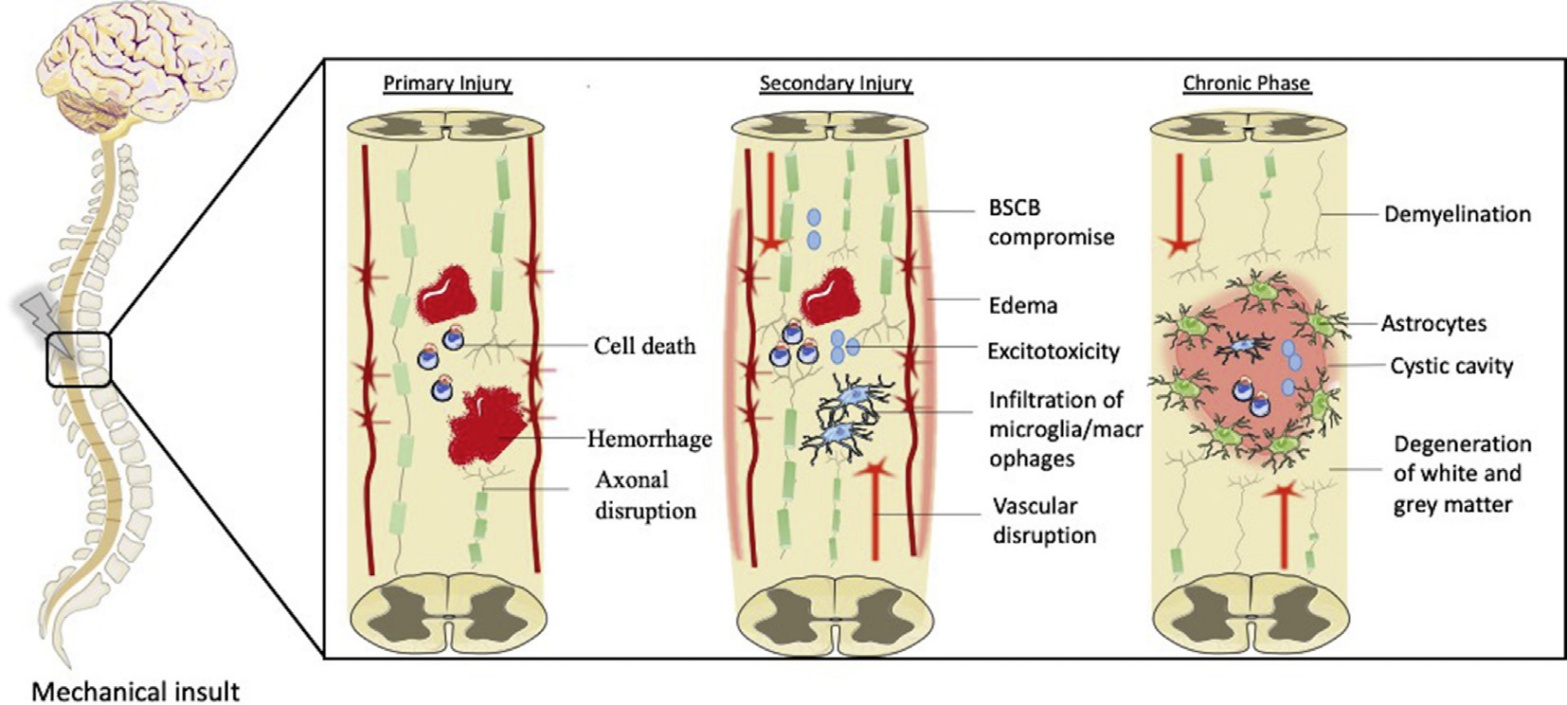
Underlying events that comprise the pathophysiology of SCI. The mechanical insult triggers a cascade of events mainly occurring at secondary phase with a massive damage of neuronal tissue. Later on, a chronic state is established, and the extremely inhibitory environment hinders tissue regeneration.

Pathophysiology of SCI comprises three phases, the primary injury caused by a mechanical insult of the bone, followed by the secondary injury and then the onset of the chronic phase (Fig. 1). Primary injury starts when spinal cord (SC) is compressed, contused, lacerated, or transected. Immediately upon injury occurs the disruption of ascending and descending pathways, beginning secondary phase, which leads to permanent neuronal damage. In addition, with the disruption of the blood spinal cord barrier (BSCB) there is the massive infiltration of inflammatory cells [14], that lead to a release of proinflammatory cytokines such as TNF-α, IL-1β, and IL-1α [15] to the extracellular milieu. Furthermore, the damage of spinal neurons, axons, and astrocytes leads to a massive release of glutamate that will bind to NMDA receptors promoting their overactivation allowing higher flow of calcium that triggers cell death and consequently death of healthy neurons [16]. Further, T cells may play a role in activation of NADPH oxidase, a protein complex that is involved in the production of reactive oxygen species, enhancing the inflammatory response and impacting in the clearance of myelin debris [17]. On top of this, the death of oligodendrocyte (OLs) precursors cells, responsible for myelination of axons, will not allow the myelination of sprouting axons [18]. Thenceforth a chronic phase is established, with demyelination of white matter and dissolution of grey matter, formation of cystic cavity due to enhancement of astrogliosis and surrounded by reactive astrocytes, microglia/macrophages and components of the extracellular matrix (ECM), particularly chondroitin sulfate proteoglycans (CSPGs) [19].

### 1.3 Current clinical approaches

#### 1.3.1 Surgical decompression

When an injured patient first arrives to the hospital there are a number of protocols that can be followed to minimize tissue degeneration and ameliorate the patient's condition. Such protocol starts right on the injury place, where proper immobilization is performed. This step alone has shown to reduce neurological deficits in 25% of the patients [20]. Once in the hospital, and after performing imaging to understand the level of injury, a surgical decompression is performed to mitigate secondary injury by ameliorating the mechanical pressure caused by hemorrhage and edema [21]. The timing to perform surgery in patients with SCI remains controversial. Although some studies defend that early surgery could improve neuronal outcomes as well as improve the score on the American Spinal Injury Association (ASIA) Impairment Scale [22] others indicate that late surgery will allow better diagnose and detection of possible other fractures and will not promote more damage to the spine [23]. Therefore, it is also important to establish a reasonable time for early and late surgery. However, it is important to notice that this could be dependent of the time that patients take to arrive to hospital and also the kind of injury that could lead to different outcomes. A study performed by Kim et al., show that 48 h is the most likely time for stabilization of the patient to go into early surgery. They also compare the early and late surgery improvements after 6 months. They show that patients with SCI, regardless the region that was affected, tend to benefit with surgery within the first 48 h after injury, either if it is a complete or incomplete lesion [24].

#### 1.3.2 Rehabilitation

Rehabilitation of SCI is a challenging process. Nowadays rehabilitation centers are specialized in non-invasive approaches, focusing in motor training using ladder walking, treadmill, swimming, bicycling, electrostimulation, or even robot-assisted training. In fact, training after injury can activate or modulate some pathways that lead to motor recovery, namely through neuro-plasticity triggered phenomena [25]. In 2008, Beaumont et al. conducted a study where they compare the recovery of lesioned rats with or without training. The training was performed between day 7 and 28 postinjury daily in their cages. They found out an increase in level of endogenous BDNF in trained animals and also a preservation of tibial motoneurons, which are important to preserve the integrity of SC [26]. Moreover, body weight-supported locomotor training in individuals with chronic incomplete SCI could improve body function/structure with better sitting and standing capability after 120 sessions with a small gain of bladder, bowel, and sexual function. Importantly, the acquired function persisted for up to 12 months, which supports the effectiveness of locomotor training for patients [27]. In recent years robot-assisted treadmill training has been introduced in rehabilitation programs mainly because it can increase the training intensity and specificity, simplify the measurement of motor improvements and automatization [28]. Pateints with incomplete SCI after robot-assisted training programs for 6 weeks showed an improvement in motor function, cardiovascular rate, mainly systodiastolic function, reduced inflammation [29], and improve respiratory function by muscle activation [30]. Furthermore, the introduction of epidural stimulation relies on the possibility of combining it with locomotor training to restore some of the function below the level of injury. For instance, patients with cervical or thoracic complete SCI were subjected to intensive training for 9 weeks, without positive outcomes. After that, electrodes were implanted epidurally and training sessions were performed with stimulation. The patients were able to walk over ground or accomplish independent stepping with weight support, using an assisted device [31]. The mechanism behind recovery after training is attributed to the natural plasticity of the CNS after injury, however other authors suggest a reorganization of spinal circuits with memory from interrupted neuronal signals [32]. In addition, innervation of serotoninergic and dopaminergic fibers were increased, oligodendrogenesis was promoted [33], as also endogenous neuronal differentiation and reconnection of some neuronal tracts [34].

Overall, rehabilitation programs have shown to be essential in motor performance recovery, despite patients are not able to walk without assistance.

### 1.4 Clinical trials

**Fig. 2:**
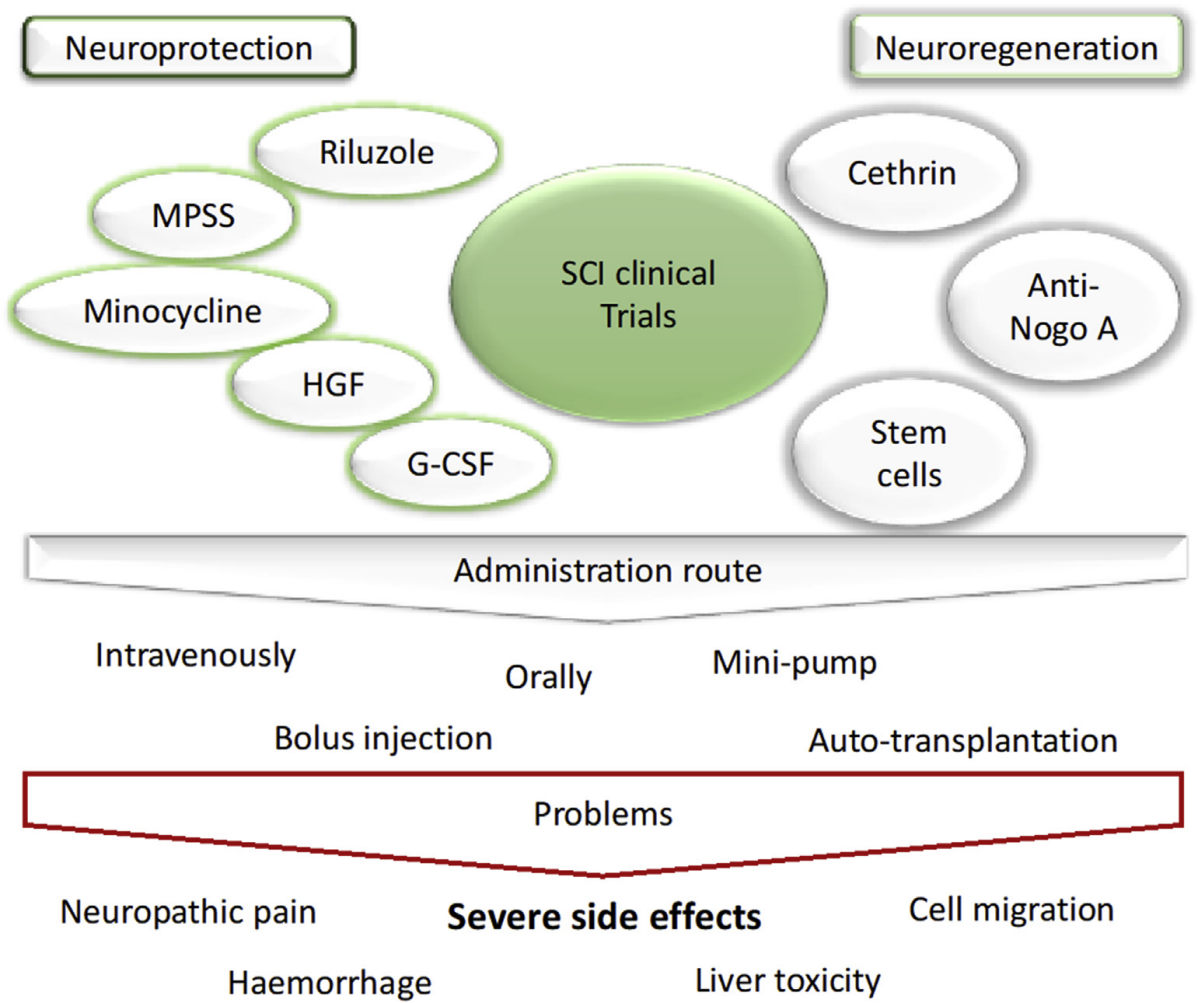
Clinical trials in SCI and related administration routs and side effects.

Animal studies have shown promising results to translate therapies from bench to clinic. The strategies are focused in reducing or inhibiting secondary injury events by neuroprotection, promoting the protection of injured tissue, as well as stop the inflammation to spread, or neuroregenerative approaches of endogenous tissue (Fig. 2).

#### 1.4.1 Neuroprotection

##### 1.4.1.1 Methylprednisolone

Methylprednisolone (MPSS) was the first drug to show promising results in promoting recovery after injury. MPSS is a glucocorticoid which act on cytoplasmic receptors upregulating anti-inflammatory factors and modulating the action of proinflammatory cytokines [21]. Beside the promising results in first clinical trial (the National Acute Spinal Cord injury Study, NASCIS I) the dose administration of MPSS was still controversial so in NASCIS II was tested an higher dose (30 mg per kilogram) by bolus injection followed by a dose of 5.4 mg per kilogram per hour in the following 23 h. They also compared the impact of administration within 8 h after injury or later. It was observed an improvement in motor function, pinprick and touch 6 week and 6 months after injury, respectively. It was also reported that the drug was more effective if administered within the first 8 h [35]. However, concerns were raised about side effects, as the administration of high doses of MPSS caused gastrointestinal hemorrhage, respiratory, urinary, and wound infections, sepsis, deep vein thrombosis/pulmonary embolism, and can lead to death [36]. Moreover, severity of side effects do not offset motor gains, which is a disadvantage for patients [37]. Owing to this situation, the recent guidelines from the Congress of Neurological Surgeons/American Association of Neurological Surgeons go against the MPSS administration in patients with SCI [38].

##### 1.4.1.2 Riluzole

Riluzole is a benzothiazole anticonvulsant approved by the U.S. Food and Drug Administration and is used to treat amyotrophic lateral sclerosis [39]. In SCI, it can modulate excitatory neurotransmitters by blocking the excess release of glutamate and sodium [40,41]. Preclinical trials deeply support the positive effects in neuronal preservation and functional recovery [42]. In phase I clinical trials, riluzole was administered orally or by nasogastric tube every 12 h. With this treatment, there was improvement in motor performance after 90 days. Moreover, there were no related side effects associated, but only an increase in bilirubin levels, that return to normal after some time [43]. This drug is now recruiting to proceed to phase II/III (Riluzole in Acute Spinal Cord Injury Study—NCT01597518).

##### 1.4.1.3 Minocycline

Minocycline (MH) is a synthetic tetracycline, used for the treatment of acne [44]. The efficacy in SCI is related with inhibition of microglial activation and proliferation, reduce excitotoxicity, reduce neuronal, and oligodendroglial apoptosis and neutralization of oxygen radicals [45]. In phase II, MH was intravenously administered in two different doses, 200 mg two-times daily and 800 mg in a rate of 100 mg every 12 h until reach 400 mg and then maintained till day 7. Despite no differences were observed between placebo and MH treated groups, there was a recovery of motor and sensory functions that plateau after 3 months, as assessed by ASIA scores. Regarding side effects, it was reported an increase in liver enzymes, stabilizing with the ending of drug administration [46]. MH is now on phase III (Minocycline in Acute Spinal Cord Injury—NCT01828203).

##### 1.4.2.4 Granulocyte colony-stimulating factor

Granulocyte colony-stimulating factor (G-CSF) factor is a hematopoiesis-stimulating cytokine that is involved in survival, proliferation, and differentiation of cells, and also the maturation of bone marrow stem cells (BMSCs) into neutrophils [47]. In SCI, studies in animal models revealed that it administration enhanced autophagy and reduced apoptosis of neurons [48]. In phase III study, a random cohort of patients with SCI were treated with 300 μg G-CSF administered subcutaneously daily up to 7 days. Patients were able to recover motor functions in ASIA scores and also improve touch, pinprick, walking, bladder, and bowel control. Side effects such as neuropathic pain, headache, nausea, and vomiting, fever, transient itching, and skin rash and upper quadrant pain were reported [49].

##### 1.4.1.5 Hepatocyte growth factor

There is an increased interest in using growth factors in preclinical therapies for SCI because they can modulate cellular functions such as intracellular communication, cell adhesion differentiation, and migration [50]. Hepatocyte growth factor (HGF) was administered intrathecally in a replication-incompetent herpes simplex virus-1 vector (rhHGF) in a contusion thoracic rat model. The results show that HGF could reduce the levels of cleavage of caspase-3 in neurons and OLs, promoting their survival and induce the regrowth of 5-HT positive fibers [51]. Considering these promising results, a phase I/II clinical trial was conducted in Japan between June 2014 and May 2018 (phase I/II Study of PK-100IT in Acute Spinal Cord Injury - NCT02193334) where patients were treated with a dose of 0.6 mg of rhHGF intrathecally 72 h after injury 5 times a week at lumbar level. The motor recovery and adverse effects, namely production of autoantibodies against rhHGF are still under analysis [52].

#### 1.4.2 Neuroregeneration

##### 1.4.2.1 Cethrin

BA-210, the commercial name for Cethrin, is an inactivator of Rho pathway that when activated can inhibit axonal regrowth, and also reduce inflammation levels [53]. In a SCI preclinical model, BA-210 reduced lesion extension, and improved functional recovery [54]. In a phase I/IIa, Cethrin was administered using a fibrin sealant placed on top of the dura mater of the SC, both for thoracic and cervical lesion. Most of the patients enrolled in this study were ASIA grade A and after treatment recovered for ASIA grade C or D. Related side effects such as urinary infections, were rarely reported [55].

##### 1.4.2.2 Anti Nogo-A

The administration of antibodies gained interest when Schwab et al. developed a therapy to deplete the effect of Nogo-A, a protein membrane that arrest the neurite growth. Antibodies strongly neutralize the Nogo-A activity [56]. Furthermore, subdural administration in injured rats showed an increase in cortical spinal tract sprouting of axons, as well as functional recovery [57]. With these promising results, later on, in collaboration with Novartis (www.novartis.com) a human anti-human Nogo-A antibody (ATI 355) was produced and started the phase I clinical trial [58]. The antibody was delivered intrathecally by continuous infusion by external pump or by bolus injection in patients with acute traumatic paraplegia and tetraplegia. This first in-human study pointed the safety and absence of immunogenic response of ATI355, minor adverse effects were reported and also some gain of neurological function either motor or sensorial [59].

In addition, another two more clinical trials are currently running to tackle Nogo-A activity. NISC – Nogo Inhibition in Spinal Cord Injury (NISCI - NC NCT03935321) in phase I/II aims to test the efficacy of NG-101 antibody by bolus injection in patients with cervical acute SCI. AXER – 204 in Participants With Chronic Spinal Cord Injury (RESET – NCT03989440) will test the effect of AXER-204, a human fusion protein that rescue the activity of myelin-associated inhibitors of axonal growth [60], administered in a single dose in phase I and repeated doses in phase II in patients with chronic cervical SCI.

##### 1.4.2.3 Stem cells

Autologous transplantation of stem cells strives in the change of the microenvironment created after lesion, assisting remyelination processes, or cell differentiation into new neurons to reconnect loss of neuronal transmission [61]. Mesenchymal stem cells (MSCs) are multipotent stem cells that can be isolated from different tissues, such as adipose tissue (adipose tissue-derived stem cells – ASCs) or bone marrow (BMSCs), from bone marrow. These cells have the capability to reduce inflammation, and differentiate in adipocytes, chondrocytes, and osteoblasts [62]. Adipose stem cells for traumatic spinal cord injury (CELLTOP – NCT03308565) phase I trial treated patients with cervical SCI with auto transplantation of ASCs by intrathecal delivery. ASIA motor score was improved as also the sensory functions after 18 months follow up. More important is the fact that patients noticed an improve of their life quality [63].

Safety Study of Local Administration of Autologous Bone Marrow Stromal Cells in Chronic Paraplegia (CME-LEM1 – NCT01909154) tested the effect of administering BMSCs in acute and chronic phase of patients with dorsal thoracic SCI. In general, was observed an improvement in neurophysiological functions, namely bladder function [64].

Herein are shown some examples of stem cells in forefront of clinical trials, other trials with different cells are currently ongoing (clinicaltrials.gov). However, some concerns remain controversial in this field, like the source of cells which is best for autologous transplantation and some ethical issues in using cell therapy.

##### 1.4.2.4 Neurospinal scaffold (INSPIRE)

In the beginning of 2000, Teng et al. [65] developed a scaffold with an inner and outer surface composed of a blend of 50:50 PLGA and a block of PLGA-polylysine. Moreover, murine NSCs were seeded in the scaffold and implanted in a thoracic hemisection injury rat model. Functional outcomes revealed improvement of motor function with frequent-to-consistent plantar stepping and coordination, reduction of glial scar with both the implant alone or seeded with cells. Later on, the implantation of this scaffold in a hemisection African green monkey model showed proof of safety, as well as some degree of recovery and neural plasticity [66]. In 2016, the first in human implantation of a bioresorbable polymer was reported. After a motocross accident, a patient was diagnosed with T11-12 dislocation fracture, and after spinal decompression the neurospinal scaffold was implanted. The patient was followed for 6 months after which improvements in sensorimotor functions and reduction of neuropathic pain was achieved. In addition, this study showed the safety and feasibility of scaffold implantation in patients with SCI [67]. This study is now in process to recruit patients for clinical trials (INSPIRE 2 - NCT03762655).

## 2 Hydrogels as delivery systems in SCI

Over the last decades, there was an increased number of clinical trials conducted in SCI. However, a considerable number failed to promote an effective recovery. Despite some promising results in promoting gain function, most therapies presented upwards, such MPSS or MH, are not already in clinics due to the high risk to trigger of severe side effects (Fig. 2). For researchers, it is quite a challenge to reduce these effects because they are mainly due to the administration route that requires the use of high doses to have a local effect, which became toxic for other organs, particularly liver [68]. On the other side, local administration is also not an option due to fast clearance by fluids. In some cases, the administration is done by mini pumps intrathecally, which is still an invasive method with some risk of infection [69,70].

In an attempt to overcome these problems, biomaterials have flourished as a promising tool in SCI therapeutic strategies. Particularly hydrogels, which are high water content crosslinking structures, with a particularity of being similar to nervous tissue. They are known of being biocompatible, have the capacity to fill the cystic cavity and support axonal growth or cell differentiation. Specifically, they can be implanted or injected at lesion site without promoting a further immune response [71]. These characteristics made hydrogels very attractive to be used as DS of drugs, growth factors, to be injected locally in a minimally invasive way for site-oriented effect avoiding the use of high doses of therapeutic agents and consequent adverse side effects [68]. Moreover, several preclinical trials have shown the accomplishment of using hydrogels as DS (Table 1).

**Table 1:** Current hydrogels delivery systems applied in SCI model and it potential to induce regeneration.

Hydrogel type		Functionalization	Therapeutic agent	Characteristics	Injury model	Delivery approach	Therapeutic effects	Reference
Natural	HAMC	KAFKA	BDNF	• Low swelling • Release for 4 days	Rat T10 clip compression	Local administration after injury	• Locomotor recovery • Inhibition of proinflammatory cytokines expression • Upregulation of anti-inflammatory cytokines	[85]
	Alginate	PLGA NPs	MH and PTX in NPs	• Irregular microporous structure • Low viscosity allowing syringe injection • MH and PTX released for 63 days	Rat T8 lateral hemisection	Local administration after injury	• Axonal regrowth • Neuroprotection mediated by reducing proinflammatory cytokines • Locomotor recovery	[154]
	Gelatin	CBD	HGF	• Photo-crosslinkable hydrogel • Furfurylamine enhanced retention of CBD-HGF into hydrogel • Release for 7 days	Mice compression injury	Intrathecal injection after injury	• Reduction of scar formation • Reduced inflammatory response • Motor recovery	[155]
	Agarose	DS-CH	BDNF	• Released over 17 days	Rat unilateral C4/C5 contusion	Local administration after injury	• Preservation of respiratory function • Enhanced diaphragm innervation • Increase 5-HT positive fibers	[144]
	Chitosan	Collagen	Serp-1		Rat T10 compression	Local administration after injury	• Improved motor function • Reduced SC damage • Reduced CD3-positive cell at injury site	[156]
		β-glycer- ophosphate/hydroxyethylcellulose	lentiviral mediated NGF-overexpressing ADSCs		Rat T8-T9 moderate contusion	Local injection one week post-injury	• Higher survival and proliferation cells at injury site • Improved motor function • Reduced cavity formation	[92]
Synthetic	Laponite	Heparin	FGF4	• Elastic modulus of 0.1 kPa • 3D porous structure • Easily injected using syringe • FGF4 released for 35 days	Rat T9 clip compression	Orthotopic injection	• Reduced cystic cavity • Axonal regeneration and remyelination • Suppression of astrocyte migration and polarization • Reduction of M1 phenotype macrophages	[157]
	Fmoc	RGD	S-220 (Epac2 agonist that could enhance axonal growth)	• Stiffness ~100 Pa • Release for 28 days	Rat T10 contusion	Local injection 3 weeks after injury	• Improve in control limb movements • Enhanced neurite growth • Suppression of astrocyte activation	[158]
	Poloxamer	Heparin	GDNF	• Gelation temperature at 37 °C • Porous structure • 80% GDNF released after 24 h	Rat T9 compression	Orthotopic injection after injury	• Neuroprotection of injured SC • Enhanced axonal repair • Inhibited expression of caspase-3	[82]
			NGF	• Porous sponge like structure • Interconnected inner porous	Rat T9 contusion	Orthotopic injection after injury	• Motor recovery • Decreased astrocytes at injury site • Increased number of CD31 positive cells • Reduced apoptosis	[159]

Although, cell transplantation is considered as a very promising approach, there is the concern of cell migration for other regions of CNS, forming ectopic colonies or triggering abnormal tissue formation [72,73]. Once more, hydrogels can be used as matrixes to retain cells locally. Furthermore, hydrogels can be functionalized with peptides, such as fibronectin (GRGDS) or a laminin motifs (IKVAV), that support cell adhesion and growth and have shown to improve recovery after lesion [71,74].

As previously referred, hydrogels appear as excellent and versatile platforms to be used in SCI therapies. More than being used as depots for drugs, growth factors or increasing the potential effect of transplanted cells they protect molecules from being degraded by enzymes or an adverse immune response as they can support axonal growth, and promote tissue regeneration while they are degraded [75]. Another advantage is that their formulation characteristics can be modulated in order to improve their performance *in vivo*, reducing further inflammatory responses as well as control drug delivery. An ideal DS will promote a burst release in the first days and a slow release for the maximum time possible. This implies that a therapeutic agent has an immediate relief upon injection, normally in the first 2–3 days, but with a prolonged effect with a continuous release, which will allow a continuous therapeutic effect without needing to perform several administrations in time [76].

Below some important characteristic of hydrogels will be highlighted in order to improve the hydrogel designing and consequently the deliver efficacy.

### 2.1 Hydrogels formulation methods and characterization techniques

**Fig. 3:**
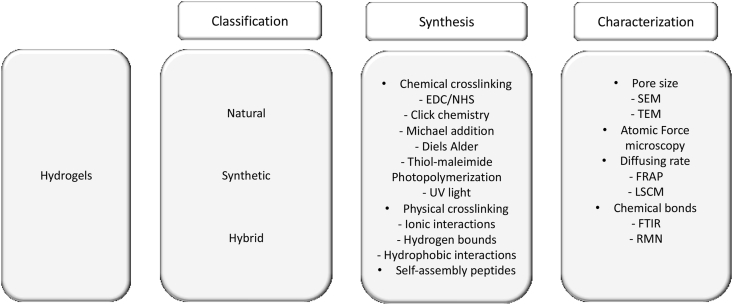
Hydrogel classification, formulation methods, and characterization techniques applied in the context of DS.

Hydrogels can be obtained from natural, synthetic sources, or formed by both natural and synthetic polymers forming a hybrid hydrogel [77]. Although natural sources have the advantage of higher biocompatibility and biodegradability, synthetic biomaterials have high water absorption, long life and higher variety in chemistry which confers them strength and resistance [71,74]. Formulation of hydrogels is of extreme importance when considering their use in context of SCI, they require outstanding improvements to enhance therapeutic effect and avoid additional surgical interventions. As an alternative injection of *in situ* forming hydrogels is a more reliable strategy. Upon injection, the fast transition from liquid to gel allows a better adaptation to the tissue at injury site, eliminating free spaces, and forming a template for tissue regeneration. Another advantage of this method is the easy formulation of hydrogels with cells, growth factors or drugs in liquid state formerly injection [78]. In this sense, several methods can be used to synthetize them, herein a brief introduction of the main processes used will be presented (Fig. 3).

Hydrogels can be physically or chemically cross-linked, depending if it is a non-covalent bond promoted by ionic interactions, hydrogen bounds or hydrophobic interactions, or if the covalent bond is formed by irradiation or a chemical crosslinker, respectively. Comparing both methods physical hydrogels have a reversible sol-gel transition and are formed by non-covalent cross-links, whereas chemical formulations have the disadvantage of using a chemical crosslinker, which can be toxic and interfere in the integrity of loading molecules [79]. However they do offer the advantage of an easy control over mechanical properties and form irreversible ligations [80,81]. As an example of chemical crosslinking EDC/NHS reaction is conducted to activate carboxyl groups of heparin for posterior formation of an amide bond between them and the amine groups of poloxamer. These hydrogels were formulated to deliver GDNF orthotopically in a compression injury rat model [82].

A wide range of chemical coupling reactions are used to synthesize hydrogels namely click reactions such as Michael type addition, thiol-maleimide reaction and Diels-Alder chemistry. The term “click chemistry” is used to characterize quick and versatile reactions, with high yield and highly selective resulting low toxic bioproducts with well-defined spatiotemporally controlled chemical network structures [83,84]. Mentioned chemical reactions allow the formation of hydrogels in water solution at physiological pH and formation of *in situ* hydrogels, which in context of SCI will help the hydrogels to better accommodate to the lesion site. Briefly, a maleimide-KAFAK was covalently bonded to methylcellulose (MC) by thiol-maleimide click chemistry for the delivery of BDNF improving neural behavior in a SCI rat model [85].

Among several other methods to formulate hydrogels, photopolymerization is used with intention to induce *in situ* polymerization controlled by photo-induced gelation by ultraviolet or visible light [78]. In this process the photoinitiator react to form a covalent bond via reacting groups. The main concern with this approach is cell viability that can be compromised by the radiation applied, mainly those with high proliferation rate. In this sense, it has to be carried out a compromise to have a fast light exposure that will promote fast polymerization [86]. Piantino et al. developed a PLA-polyethylene glycol (PEG)-PLA triblock copolymer that are assembled by photopolymerization of methacrylated (MA) groups of the macromers for delivery of NT-3. Upon injection, hydrogel was exposed to light for 60s, an adequate time to induce polymerization and prevent dispersion of the hydrogel from the injection site [87].

Another interesting strategy is the use of self-assembly peptides. This strategy allows the formation of microbuilding blocks rationally and coherently into a tissue-like assembly, more likely synthetic amino acids based molecules that have a fast sol-gel transition at neutral pH. Moreover, self-assembly is a natural phenomenon used to construct complex structures from simple building blocks. For instance, microgels can be induced by magnetic, acoustic, mechanical, capillary forces, surface tension, or polarity [86,88].

Considering hydrogel characterization, it is crucial to study their structure and functionality. For that purpose, several microscopical techniques are the gold standard to explore hydrogel structure. Scanning electron microscopy (SEM) is by far the most popular technique used to perform morphological characterization of hydrogels. In this context, SEM allows the identification of pore formation and size, crosslinking status of a hydrogel and evaluate the effect of loading molecules in general structure of matrices [89]. Although SEM is widely used, it has a limitation capacity of generation two-dimensional projections. To overcome this limitation, laser scanning confocal microscopy has been introduced in the field. In addition, LSCM can be combined with fluorescent dyes, such as fluorescent recovery after bleaching (FRAP) to study the diffusivity from hydrogels, which will contribute to predict the release profile in DS [89,90]. Spectroscopic methods include Fourier transforms infrared (FTIR) spectroscopy is an analytical method used to study the bonding structure of atoms based on the interaction between infrared irradiation with matter [91]. It can be used to characterize reactions between specific chemical groups such as chitosan and β-glycerophosphate [92].

### 2.2 Rheology

Mechanical properties of hydrogels are of the utmost importance when they are considered for implantation. Rheology is a process where the hydrogel is subject to small deformation, normally small amplitude oscillatory shear measurements, in a rheometer where is measured the deformation energy stored during the shear process (G′) also mentioned as elastic modulus or stiffness, and the energy dissipated during the process (G″) also referred as viscosity. Hydrogels are more elastic if G′> G″ or are more viscous if G″ > G′ [93]. In 2010, Lampe et al. investigated the effect of different macroscopic properties of PEG hydrogels in the growth and differentiation of neural progenitor cells (NPCs), in which by increasing the amount of polymer in solution, hydrogels ranging from of 1 kPa–20 kPa elastic modulus were obtained. In 3D models, the increased stiffness hindered the metabolic activity of cells leading to a higher apoptotic rate. Noteworthy, with increased polymer concentration the access to nutrient could be hampered by the reduced mesh size, which could explain cell apoptosis [94]. In line with this finding, hyaluronic acid (HA) hydrogels were modified with increased MA groups ratio to increase the hydrogel stiffness. Elastic modulus varied between 3 kPa and 5 kPa with increased MA substitution. Notably, neuronal differentiation was better supported by soft hydrogels, in which more mature neurons and increased axonal length were detected. Surprisingly no effect of mechanical properties was noticed in the morphology of astrocytes isolated from SC [95].

Later on, Schultz et al. [97] developed a DS using a very soft agarose hydrogel with approximately 1.5 kPa. In this work, they aimed the release of thyroid hormone 3,3′,5-triiodothy-ronine (T3) to tackle the enhancement of OLs at injury site favoring the myelination of sprouting axons [96]. The release of T3 in a unilateral cervical contusion injury rat model increased the number of newly formed mature OLs and consequent axonal myelination rostral to lesion site.

In a different approach, hydrogels can be used in a strategy to decrease the mechanical properties of scaffolds. The combination of collagen in a scaffold of poly (propylene fumarate), allow the formation of a porous structure filled with collagen for the development of a DS. Furthermore, collagen ameliorated the release of neurotrophin-3 (NT3), previously combined with collagen binding domain (CBD) which binds to biomaterials and can retain high content of neurotrophic factors, for 7 days. In a complete SC transection at thoracic level, the scaffold surpasses organized axonal growth throughout the porous structure. The incorporation of collagen improved the production of mature neurons and NT3 enhanced survival of endogenous NPCs [98].

Beyond elastic modulus, viscosity of hydrogels must also be considered. It is related with the capacity of hydrogels to be injected through a needle and can diffuse before gelation [99]. Heparin poloxamer (HP) was used to deliver bFGF at a thoracic SCI hemisection. The hydrogel viscosity ranged between 8 Pa s to 10 Pa s as the temperature change from 32 °C to 37 °C [100]. Imidazole-poly(organophophazenes) polymer hydrogel (I-5) was implanted in a thoracic contusion injury rat model. This hydrogel has the capacity to rapidly form a gel-like material with a viscosity of 50 Pa s and in just 150s achieving a viscosity of 600 Pa s at body temperature. *In vivo*, its implantation almost eliminated cystic cavity and promote neuron repair and motor recovery [101].

Accordingly, hydrogels to be used in SCI therapies should mimic the mechanical properties of host tissue to allow the regenerative processes to take place. In agreement with literature, the stiffness of SC could range between 3 kPa and 300 kPa [102,103] which means that hydrogels have to be stiff enough to assemble itself and soft enough to create an appropriate environment for cells to growth, adhere and differentiate. Moreover, it was shown that soft hydrogels (<1 kPa) with low viscosity are suitable for SCI implantation to favor tissue regeneration [104].

### 2.3 Mesh size

Hydrogels are polymer networks that form a gel when exposed to a polymerizing agent promoting the crosslinking between polymer chains. The free space between two points is named as mesh or pore of a hydrogel. Depending on the distance between the entangled points, hydrogels could be classified as macro-porous, microporous or non-porous [105]. Mesh size can be modulated by polymer and crosslinker concentration or external stimulus as pH or temperature. The porosity of hydrogels is crucial for delivery of therapeutic agents in injury because in most cases this process occurs by diffusion or by interactions between polymer and loading agent. If the pores are smaller than the size of therapeutic agent, it will be entrapped into the hydrogel and will not be released. On the other hand, if the hydrogel pores and molecule size are similar the result will be a slow release. Contrarily, if pores are higher than the agent, small molecules will move freely in the network and release will occur by diffusion, release is not directly correlated with mesh size [106]. Recent developments have been made to determine mesh size. Among them, FRAP bleaching is used to study the diffusivity of fluorescent molecules from hydrogels. This technique has the advantage of being performed in a common confocal laser scanning microscope with a fluorescent probe capable of photobleaching [90]. Fluorescence correlation spectroscopy is a complement to FRAP, once it correlates diffusivity coefficients from resident times of fluorescent particles moving through a small, static illuminated volume, being ideal for microscale heterogeneities in the hydrogel structure. This method has the advantage of using less powerful laser than FRAP, as well as low fluorophore concentrations [107].

Currently, the most effective technique to determine mesh size (ξ), is correlating with swelling data, a theory of Canal and Pepas, Eq. (1) [108,109].

(1) $$\xi \;=\;\phi^{-\frac{1}{3}}\;{\left(\left(1\;-\;\frac{2}{f}\right)l^{-2}C_{\infty }\frac{\lambda M_{c}}{M_{r}}\right)}^{\frac{1}{2}}$$

where, ϕ is polymer volume fraction, l polymeric carbon-carbon bond length, C_∞_ is the polymer-specific characteristic ratio for a chain of ∞ repeating units, λ polymer backbone bond factor, M_c_ number of average molecular weight between cross-links in a polymer network and M_r_ molecular weight of the polymer repeating unit.

Porosity in network structure is also imperative for diffusion of nutrients within the lesion site or even to allow the migration of cells or axons to growth through the implanted hydrogel. Chen et al. developed a macroporous hydrogel based on 2-hydroxyethil methacrylate (HEMA) with MOETACL to deliver bFGF at injury site. This network was favored by communicating pores with an average size of 80 μm. In a complete thoracic transection, the implantation of this DS allowed the recovery or motor performance evidenced by the increase ingrowth of axons and blood vessel in the hydrogel only 5 days after injury [110].

In the field of DS, the possibility to modulate pore size is of extreme importance, considering also the injectability of the matrix. In this sense, the formulation of matrices as DS using an oil-in-water emulsion at different ratios and surfactant concentrations allows the control over porous structure. Briefly conjugation of both methods allows *in situ* pore formation by incorporation of oil droplets within crosslinkable precursor solution and polymerization induced by UV light, upon immersion in water the diffusion of oil droplets results in pore formation. Pore size was of the oil droplet size but with increasing concentration of surfactant pore size diminish [111].

In a more simple and easy way, pore size can be tunable by controlling crosslinking degree, namely increased crosslinking degree or hydrogel precursor content decrease mesh size in a matrix hydrogel [112].

### 2.4 Swelling

Swelling behavior of the hydrogels is the process in which water molecules will enter in the structure of the polymer promoting the expansion of mesh size which will allow more water molecules to enter. In this process, elasticity offset the extreme expansion of the network, avoiding it destruction. Thus, the equilibrium is reached when there is a balance of these two forces, also known as swelling pressure (P_sw_), is equal to zero. Generally speaking, swelling takes in account the increase in weight, volume, or length of an hydrogel and can be measured as degree of swelling (D_sw_), Eq. (2) [113].

(2) $$D_{sw}=m_{hw}/m_{hd}$$

where m_hw_ and m_hd_ is the weight of wet and dry hydrogels, respectively. It could also be measured by the diameter of swollen hydrogels, Eq. (3) [114].

(3) $$D_{sw}={\left(d_{hw}/d_{hd}\right)}^{3}$$

where d_hw_ and d_hd_ are, respectively, diameter of hydrogels after and before swelling, which always assume values D_sw_ ≥1 [113].

After SCI, there is an increased pressure in surrounding tissues responsible by the edema formation, which causes cell death and tissue loss [99]. Considering this, hydrogels to be used in therapeutic strategies should not hold a high degree of swelling to avoid more pressure in the site of implantation [99]. Moreover, it is also important to control the swelling behavior to control the release of molecules. Hydrogel swelling is directly correlated with porous structure, so all strategies to modulate mesh size mentioned ahead influence swelling behavior. Hydrogels with small pores will swell very slowly while macroporous structures swell fast [115].

For instance, the increase of crosslinking density of HA-tyramine conjugate by increasing the concentration of H_2_O_2_ will decrease the swelling ratio and therefore decrease the rate of protein release [116]. Besides, swelling can also be controlled by the thiolation process of chitosan, that when modified with Traut's reagent immediately swell after chitosan and PEG precursors are mixed and form a gel, slightly shrinking. Such approaches can reduce the likeliness of hydrogels to swell in the injury site, avoiding adverse reactions such as inflammation [117].

Similarly, hydrogel swelling can be modulated by incorporation of thermoresponsive segments in a hydrogel matrix. They have the particularity of being sensitive to thermal stimuli. Basically, these segments are water soluble and at low temperatures they are hydrated due to interactions between water molecules and hydrophilic domains extending polymer chains. On the other hand, high temperatures promote their dehydration and polymer chains aggregate due to hydrophobic interactions. Briefly at low temperature hydrogels swell while at high temperatures the swelling is slow, so hydrogel swelling can be controlled by combining hydrophilic and hydrophobic components [118]. Such polymers are poly(ethylene glycol)/poly(propylene glycol), poly (glycidyl ethers) cellulose derivates, poly (N-substituted acrylamide) [[119], [120], [121]]. The main advantage of this strategy for injectable hydrogels is that hydrogels will polymerize upon injection normally at body temperature.

### 2.5 Degradation

Introduction of biomaterials in regenerative strategies for CNS imply that progressively after implantation they will degrade as axons regenerate. This is extremely important to avoid inflammation or nerve compression. Accordingly, despite some promising results with synthetic nondegradable materials, such as silicone, polyacrylonitrile/polyvinylchloride, poly(tetrafluoro-ethylene), and poly (2-hydroxyethyl methacrylate), their use is not frequent due to their non-biodegradability. On the other hand, most of degradable materials used are provided by natural sources or synthetic materials, such as PLGA, PLA, PGA, or PEG also used in medical devices [122].

Network degradation plays an important role in controlling molecular release, this is, controlling hydrogel degradation alter release of therapeutic agent. Piantino et al. [87] controlled the release of NT3 by modulating the number of degradable units and macromer concentration in an acrylated PLA-b-PEG-b-PLA polymer. This hydrogel is formed by the addition of degradable lactic units of hydroxyl groups. NT3 had a burst release in the first 24 h, being slowly released thereafter for periods up to 2 weeks. This sustained release in a thoracic SCI transection promoted recovery of motor function as plasticity of raphespinal tracts. Hydrogel degradation can also be modulated by crosslinking with specific sequences that will be recognized for endogenous or implanted enzymes. For instance, a HA hydrogel crosslinked with MMP-sensitive peptide was tested in a thoracic compression injury rat model for BDNF delivery. When implanted *in vivo*, the endogenous cells secrete MMPs that degrade the sensitive peptides and consequently promote the release of BDNF, which induce motor neuron regeneration and motor function recovery [123]. Delivery of BDNF was also performed using a peptide amphiphilic hydrogel. In this work, a hydrogel was functionalized with IKVAV. The particularity of this hydrogel is that when implanted *in vivo*, it will assemble in nanofibers and BDNF release is controlled by electrostatic interactions. However, ultimately the release is controlled by hydrogel degradation once nanofibers will get shorter and mesh size will increase. This explains the release profile with a burst release in the first 3 days, followed by a slow release until 21 days after implantation. In a thoracic compression injury rat model, such approach preserved axonal degeneration and diminish astrogliosis [124].

### 2.6 Gelation temperature

The gelation timing of a hydrogel to be used in DS is crucial to determine the therapeutic effect of loading agent. The gelation temperature (GT) is determined when elastic modulus is the halfway for gel formation [125]. Hydrogels can also be responsive to stimulus, in this case can gelate in response to temperature. The ideal behavior is that hydrogels are liquid at room temperature, and gel at body temperature, *in situ* gelation. This will ensure that hydrogel could be injected in the lesion site using a needle and, once there, a fast gelation process will allow prolonged therapeutic effect avoiding wash away by CSF [126]. One example of thermoresponsive hydrogels is poloxamers, that are composed of a triblock with a central hydrophobic block of polyoxypropylene (PPO) flanked by two hydrophilic blocks of polyoxyethylene (PEO) [127]. Liu et al. investigated the impact of polymer concentration on GT. They formulated hydrogels with different ratios of PPO:PEO, Pol-1(PEO_101_-PPO_60_-PEO_101_) and Pol-2 (PEO_88_-PPO_27_-PEO_80_), which form a gel at 15 °C and 50 °C, respectively. Interactions between hydrophilic PEO and hydrophobic units will determine the GT and nature of hydrogel. None of the created hydrogels were suited for implantation, so they combine both and investigated the GT by varying their concentrations and found out that forming a hydrogel with 5% (w/w) Pol-2 and 17,8% (w/w) Pol-1 the GT was around 39 °C. GT was determined by varying G′ and G″ as function of temperature and heating rate of 1 °C min^−1^. Furthermore, it was used to deliver monosialoganglioside (GM1) in a T10 dorsal hemisection rat model. The loading of this molecule decreased the GT for 36 °C due to the presence of two long fatty acids, which reduce the ratio of hydrophilic and hydrophobic units in the hydrogel. GM1 was released for 1 month and inhibited the formation of glial scar enhancing neuron regeneration [128]. Poloxamer are also functionalized with heparin to delivery bFGF. These hydrogels have the ability to undergo from liquid to gel transition in about 100s as the temperature increases to 34 °C. More importantly, loading bFGF does not change the gelation properties of hydrogel. In a T9 hemisection model induced axonal regeneration, apoptosis decrease and recovery of motor function [100].

### 2.7 Surface charge

The physical and mechanical properties of the hydrogel surface also influence cell adhesion or axonal growth. The coating of surfaces with positive materials enhance cell adhesion and growth [129]. Hejcˇl et al. investigate the impact of HEMA hydrogels with different charges in thoracic hemisection injury rat model. They designed HEMA hydrogels with negative charge (hydrogel crosslinking with methacrylic acid in sodium salt (MA^−^)), positive charge (crosslinking with MOETA^+^) and a neutral polymer with positive and negative charges combined by crosslinking with both agents used before. The growing connective tissue was favored by the positive charges with the pores of the hydrogel being filled by it with evident longer neurites growing throughout the polymer with positive functional groups, which is not found in polymers without charge. On the other hand, astrocyte ingrowth was only detected in the periphery of negatively charge implant and also in uncharged polymer [130]. To overcome the negative effect of negatively charge alginate hydrogels in cell adhesion or axonal growth, hydrogels can be coated with positively charged poly-amino acids and laminin. *In vitro*, neurite growth was more pronounced in hydrogels coated with poly-l-ornithine/laminin and absent in uncoated hydrogels. In a cervical hemisection, rat model more neurites growth through the hydrogel in rostral zones and also more infiltration of host cells [131].

## 3 Strategies for delivery control

### 3.1 Affinity-based systems

The efficacy of a therapeutic strategy relies on the targeted events and the time window in which it will have the best regenerative influence. Having this in mind, besides controlling hydrogel network, establishing interactions between drug and polymer network could be crucial for sustained or on-demand release. This led to the development of affinity-based systems, in which drugs are attached to the hydrogel by covalent conjugation or through secondary bounds such as electrostatic interactions, taking advantage of protein's natural affinity for specific ligands [106]. HA/MC hydrogels were modified with a Src homology 3 domain (SH3) that were bound in the MC hydroxyl groups to improve release of chondroitin ABC (ChABC) which has thermal instability [132]. SH3 peptide binds reversibly to SH3 protein and once expressed slow the protein release. Moreover, by combining with ChABC the release rate is controlled by the ratio between SH3 peptide and SH3 protein for an *in vitro* release for 7 days [133]. In a compression injury rat model, ChABC treated animals have a decrease in CSPG levels and wide distribution of NPCs in the SC [134]. After the affinity-based systems, the incorporation of heparin in poloxamer hydrogels increases the affinity for proteins, growth factors, cytokines by binding to hydrogels through –COOH or –OH and proteins with –SH groups to extend the release. Moreover, these hydrogels are thermosensitive with a controlled sol-gel transition temperature with porous structure, which favors the delivery. HP hydrogels were used to deliver aFGF in a compression injury rat model. The release of aFGF was improved with heparin, being released about 55% after 28 days, while in hydrogel without heparin the release was about 25% of loaded aFGF. This promoted protection of BSCB, improved remyelination, reduced neuronal death contributing for motor recovery [135]. Besides enhancing drug delivery HP hydrogels do not have the capacity to store or stabilize growth factors once they are released, compromising it role in regeneration. To overcome this, hydrogels are combined with decellularized SC extracellular matrix (dscECM) that is crucial for cell adhesion, growth factors support, cell growing and survival [136]. Xu et al. incorporated dscECM loading bFGF in HP hydrogels. In this system, bFGF was released by affinity from dscECM and then by diffusion through porous structure of HP, which resulted in 25% of loaded bFGF still remained in hydrogel after 7 days. The implantation in an hemisection injury rat model promoted a reduction in cell apoptosis, neuronal regeneration contributing to motor function recovery [137].

### 3.2 Incorporation of nanoparticles

Furthermore, to protect a therapeutic agent from degradative enzymes or to enhance delivery, hydrogels are combined with nanoparticles (NPs). This hybrid system aimed to recapitulate the specificity of bolus injection with sustained release offered by mini pump or catheter but avoiding further tissue damage. Nanoparticles are used as carry and delivery agents while hydrogel ensures they are localized in the injury site to promote regeneration [75]. PLGA NPs are widely used in SCI due to its good biodegradability, and it incorporation in HAMC hydrogels allow the formulation to deliver NT-3 a modulator of maintenance, proliferation and differentiation of neurons [138], in a thoracic compression model. NT-3 was released for 28 days *in vivo*, and the release is mediated by electrostatic or hydrophobic interactions between NT-3 and PLGA NPs. This resulted in an increased axonal density surrounding the injury site with a slightly improved motor function [139]. A similar effect was observed when using the same system to co-deliver NT-3 and anti-NogoA, for 28 days and 10 days, respectively. Moreover, the author suggest that co-delivery of NT-3 can enhance Anti-NogoA effect resulting in both tissue and motor improvement [140]. Chitosan is a natural positively charged polymer often used in the formulation of particles in SCI therapeutic strategies due to its biocompatibility, biodegradability and low toxicity, antitumor activity and low immunogenicity [141,142]. A regenerative effect was observed when chitosan particles loaded with NT-3 were embedded in collagen hydrogel, and used in the treatment of thoracic hemisection monkey model, where increased axonal growth and revascularization were observed at injury site [143]. Furthermore, chitosan can be used to form particles or self-assemble with other materials such as dextransulfate (DxS) to deliver BDNF. In this context was observe a preservation of diaphragmatic function as well as enhancing serotonergic enervation of phrenic motor neurons (PhMNs) [144].

Chitosan has the advantage of being a soft material, has a higher water content and viscoelasticity, which recapitulate the mechanical properties of SC tissue. Moreover, structurally is similar to glycosaminoglycans, one of the most abundant polysaccharides of ECM [145,146]. All these characteristics made chitosan very attractive to use in SCI.

Other organic polymers, such as liposomes, have been also combined with hydrogel for co-delivery of docetaxel (DTX), a microtubule-binding agent important for growth cone development [147], BDNF and aFGF. Wang and colleagues developed a scar-homing liposome to load BDNF in the inner aqueous phase and DTX in lipid membrane modified with a tetrapeptide (cystine-alanine-glutamine-lysine) (CAQK) that binds to CSPGs. The liposomes were embedded in HP hydrogels loading aFGF. Importantly, sol-gel transition occurs at 24 °C, still suitable for injection and polymerization *in situ*, and a porous sponge-like structure with interconnected pores allow the diffusion of liposomes to release the loaded molecules at injury site, being DTX, BDNF and aFGF sustainably released for 10 and 21 days, respectively. *In vivo*, in a thoracic contusion SCI model, the combinatorial therapy contributed for mild SC lesions, increase of molecules responsible for axonal remyelination, such as MBP, induced microtubule stabilization essential for growth cone maintenance favoring axonal growth, leading to locomotion improvements [148].

### 3.3 Ion-mediated interactions

As mentioned previously, MH is a promising candidate to treat SCI; however its administration is still a challenge, for one hand systemic administration gives rise to multiple side effects, on the other side loading into hydrogels is still difficult once MH is water soluble, which means it will be released very fast and is also fast degradable in solution at body temperature [149]. The formulation of DxS-MH complexes self-assembled by metal ion binding-mediated interaction allow sustained release of MH [150] that was loaded in agarose hydrogels to ensure local delivery. Moreover, Mg^2+^ allow the formation of insoluble complexes between DxS and MH, being it release mediated by strong metal ion-mediated interactions between DxS and MH. This complex combination allows the modulation of MH release by reducing the ratio of DxS/MH and MG^2+^ concentration or by adding chitosan that compete with MH to bind to DxS and Mg^2+^. In this way, MH has a high dose being released at acute phase to tackle the primary inflammatory events, and a low dose sustainably released to hindering chronic inflammation and OLs death. Its administration in a unilateral cervical injury model was more effective than intraperitoneal injection in reducing cystic cavity, was able to reduce inflammatory response and contribute to motor recovery [151]. Similarly, MH delivery preserved diaphragm innervation of PhMNs axons, protecting respiratory circuit from degeneration and hampered further inflammation by inhibiting M1 polarization [152].

## 4 Conclusions and future perspective

**Fig. 4:**
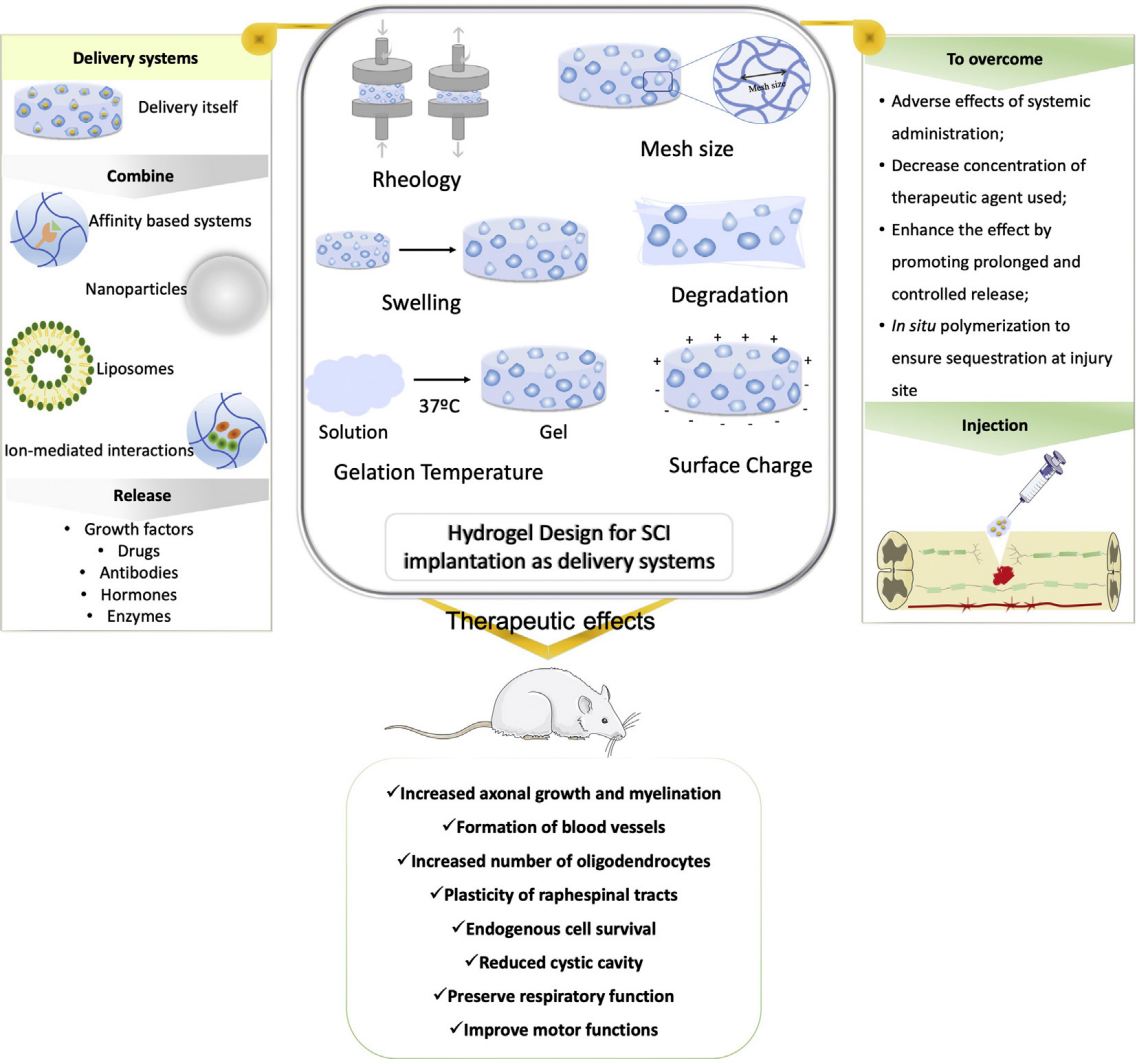
Hydrogels have been applied in SCI as delivery systems to improve the therapeutic effect of loading agents. Characteristics as rheology, mesh size, swelling, degradation rate, GT or surface charge, illustrated in the center of figure, can be modulated in order to improve their performance when injected at injury site. Additionally, hydrogels can be combined with other molecules to deliver agents at and extended time. Among all, growth factors, drugs, antibodies, hormones, or enzymes are the most frequent therapeutic agents loaded in hydrogels in SCI. In preclinical models of SCI, these systems have shown promising impact in promoting tissue regeneration.

SCI affects both motor and sensorial functions of millions of people worldwide being a serious social, economic and emotional problem. Despite the growing research in the field with multidisciplinary approaches raising to promote regeneration there is still a lack of an effective therapy in the clinic that could improve life quality of patients. Biomaterials can represent a good alternative to apply as DS, overcoming side effects reported in several clinical trials. As discussed, it is possible to modulate their designing characteristics to improve its performance at injury site and support regenerative events promoted by delivered molecular therapies. In preclinical trials, the use of these systems has allowed preservation of respiratory functions reduction of inflammation, enhanced axonal regeneration, reducing cellular apoptosis leading to recovery of motor function (Fig. 4). While all reported strategies are relevant when considering the design of effective DS some can be considered most favorable to further explore, mainly due to easy chemistry used or simplest modulation of important characteristics. For instance, it is clear that the modulation of characteristics such as stiffness, mesh size, swelling and GT have in common alterations of crosslinking degree achieved by altering hydrogel precursor content. In this context poloxamer hydrogels appear to easily modulate these characteristics. Moreover, they have the advantage of being thermoresponsive hydrogels undergoing sol-gel transition at body temperature. In addition, the possibility of incorporating molecules for affinity-based release, such as heparin, is advantageous once is based on natural affinity for proteins or growth factors, prolonging the release profile and taking also advantage of heparin being a glycosaminoglycan present in ECM.

Notwithstanding the promising results achieved in preclinical trials there is still a big question related to biomaterials, that why so few of them have reached clinical trials. The long path from bench to clinics comprehends the regulatory approval, which often tends to be quite challenging for strategies dealing with scaffolds [153]. Moreover, production of biomaterials for human implantation has to ensure Good Manufacturing Practices, as well as low batch to batch variability of natural or synthetic materials, a fact that is also challenging when scaling up lab run processes to an industrial scale [76]. Upon overcoming all mentioned problems, another concern is the injection method, which needs to be fast and low invasive, do not promote further damage as well as protecting all therapeutic agents within the formulation.

As mentioned most of the strategies to test hydrogels are conducted in transection models because of the gap created by the lesion that will allow the hydrogel to fill the cavity without fostering pressure to surrounding tissue. However, most human SCI are contusions/compressions, so another facing problem for clinical translation is the injection of biomaterials in these conditions.

Upon several developments in SCI therapies, there is a strong know-how about its pathophysiology, which is crucial when developing effective therapies. Taking this into consideration, the development of a therapy will benefit from a multidisciplinary strategy of integrated knowledge of several areas, namely biology, physics, medicine, engineering, and bioengineering and material science to try to tackle all degenerative events. Hydrogels can help with the adhesion of cells, their differentiation in neurons or uphold axonal growth, while at the same time have the ability to release growth factors or drugs that will reduce inflammation, induce axonal growth or decrease tissue damage. Similarly, materials with electrical properties could be embedded in hydrogels, as well as the combination with stimulation will favor tissue regeneration and improve patient's rehabilitation. Altogether, overcoming these problems will contribute to improve patient's life quality.

## Declaration of competing interest

The authors declare that they have no known competing financial interests or personal relationships that could have appeared to influence the work reported in this article.
